# Small Data and Its Visualization for Diabetes Self-Management: Qualitative Study

**DOI:** 10.2196/10324

**Published:** 2019-08-13

**Authors:** Sally Jane Burford, Sora Park, Paresh Dawda

**Affiliations:** 1 News & Media Research Centre Faculty of Arts and Design University of Canberra Canberra Australia

**Keywords:** mobile health, type 2 diabetes, health data, mobile tablet devices, self-management

## Abstract

**Background:**

As digital healthcare expands to include the use of mobile devices, there are opportunities to integrate these technologies into the self-management of chronic disease. Purpose built apps for diabetes self-management are plentiful and vary in functionality; they offer capability for individuals to record, manage, display, and interpret their own data. The optimal incorporation of mobile tablets into diabetes self-care is little explored in research, and guidelines for use are scant.

**Objective:**

The purpose of this study was to examine an individual’s use of mobile devices and apps in the self-management of type 2 diabetes to establish the potential and value of this ubiquitous technology for chronic healthcare.

**Methods:**

In a 9-month intervention, 28 patients at a large multidisciplinary healthcare center were gifted internet connected Apple iPads with preinstalled apps and given digital support to use them. They were invited to take up predefined activities, which included recording their own biometrics, monitoring their diet, and traditional online information seeking. Four online surveys captured the participants’ perceptions and health outcomes throughout the study. This article reports on the qualitative analysis of the open-ended responses in all four surveys.

**Results:**

Using apps, participants self-curated small data sets that included their blood glucose level, blood pressure, weight, and dietary intake. The dynamic visualizations of the data in the form of charts and diagrams were created using apps and participants were able to interpret the impact of their choices and behaviors from the diagrammatic form of their small personal data sets. Findings are presented in four themes: (1) recording personal data; (2) modelling and visualizing the data; (3) interpreting the data; and (4) empowering and improving health.

**Conclusions:**

The modelling capability of apps using small personal data sets, collected and curated by individuals, and the resultant graphical information that can be displayed on tablet screens proves a valuable asset for diabetes self-care. Informed by their own data, individuals are well-positioned to make changes in their daily lives that will improve their health.

## Introduction

### Background

It is commonplace for individuals to supplement their healthcare professional’s advice with information that is widely available on the web. The web presents a vast repository of detailed health information that is available to people with chronic diseases, and much of that information is presented as text and static diagrams and is supportive of the self-management of disease [1]. It informs its consumer about symptoms, treatments and prognoses of disease, while varying in accuracy, authority and usefulness. Global information retrieval systems, such as Google, assist in locating specific health information [1].

The mobile phenomenon, initiated by the prolific uptake of smartphones and mobile tablet devices in tandem with expanding wireless networks, creates new opportunities for individuals to contribute to the management of their own health. The mobile device provides an access point to health information in new and varied contexts [2]. It also presents opportunities in the form of the proliferation of mobile apps developed for healthcare and made available by services such as the iTunes store [2].

Embedded in apps is the potential for personal data storage, modelling and presentation for health self-management. Apps with embedded algorithms to collect, manage, analyze and present small, personal, health data sets are widely available at little or no cost to the user. Isolated, small data sets are simple to curate and to manipulate. In contrast, attention to the phenomenon of big data is prominent in society currently. Big data refers to data collections that are vast and complex. It arises in health contexts as the aggregation of data from large populations, which is then used to establish trends, outcomes and predictions.

This article reports a longitudinal mobile health intervention for people with type 2 diabetes and reveals new ways in which individuals can use mobile technology for the self-management of their health. Discussed within is the use of personal health data recording, modelling, and depictions in dynamic diagrams, charts and graphs to support the self-management of disease.

### Previous Studies

The World Health Organization’s Global Observatory for eHealth provided a definition of mobile health as:

medical and public health practice supported by mobile devices, such as mobile phones, patient monitoring devices, personal digital assistants (PDAs), and other wireless devices [3].

Mobile health was first enabled by the smartphone, its functionality and corresponding apps. A decade of research [4,5,6] positioned the smartphone as a valuable device in healthcare initiatives that encourage weight loss, smoking cessation and depression treatment.

More recent studies expanded mobile health to include the use of mobile tablet devices. Zarghom et al assessed the usability of the touchscreen of tablet devices for patients in a Canadian family health clinic and reported that 94% of all participants regarded the device as easy to use [7]. Hunt, Sanderson and Ellison explored the use of iPads in type 2 diabetes self-management and required 17 participants to log their self-management behaviors [8]. They found that an individual who logged their behaviors using iPad apps had an increased awareness and participation in self-management [8]**.** The healthcare professional is also helped when a tablet device is embedded in their work, especially when that work is traditionally mobile in its nature [9].

### Self-Management and Empowerment

Individual self-management of diabetes is desirable from a national health care perspective. Recognizing the burden that diabetes presents to the country, a recent Australian consultation paper to the government recommended a strategy of reduction of complications by increased emphasis on people with diabetes being active participants in their own care [10]. To maximize their health, self-management is important to an individual with type 2 diabetes. Coyle, Francis and Chapman outlined some of the activities of self-management, which include blood glucose testing, taking medication, and following a regular eating and exercise plan [11]. They also noted the need for individuals to be knowledgeable about their disease and respond capably to problems. Funnell and Anderson claimed that for successful outcomes, “a self-management plan has to fit patients’ goals, priorities, and lifestyle as well as their diabetes” [12] and that pre-determined health care plans cannot accommodate the complexity of daily life and the myriad decisions that must be made.

Self-management is accompanied by a model of care that moves away from a traditional medical model based on acute, symptom-driven approaches [12]. Meetoo and Gopaul described an empowerment model of healthcare in which:

people with diabetes are empowered when they have the necessary knowledge, skills, attitudes and self-awareness to influence their behaviour and that of others in order to improve the quality of their lives [13].

While health care professionals are able to create a climate in which a patient desires to be empowered, it is always an individual’s choice to become empowered [13,14]. Menon holds that individual empowerment is an experience of power and that having power leads to an experience of a sense of control [15]. Samoocha et al established the potential of the internet and eHealth to provide empowering opportunities for patients to make choices and take charge of their health [16], while Burford et al applied health locus of control theory to the intervention reported in this article and reported an increased sense of responsibility for health among research participants [17]. Gimbel et al used the construct of patient activation to design a study that levered mobile technologies for the self-management of type 2 diabetes [18]. They described activated patients as motivated, confident, skillful, and capable of changing their behavior [18].

### Collecting and Using Health Data

Mayer-Schonberger and Cukier reflected on the phenomenon of big data, noting that the scale of digital data that human society has collected and stored has resulted in the potential to “extract new insights or create new forms of value” [19]. They emphasized that big data is not just about the volume of data, but about patterns and predications that can be revealed [19]. Big data is found in many contexts, including the healthcare sector. Keen contested the increasing build of data in healthcare, suggesting that a centralized “bureaucratic data processing model” [20], such as national electronic patient records, is at odds with frontline, distributed and networked health care services. Keen noted that for health, big data processing is favored over more agile systems that might better serve the health care professional and the nature of their work [20].

The Finnish government offered a blueprint, MyData, to shift control of personal data away from businesses and governments and to place it in the hands of the individuals who are the subjects of the data. MyData idealistically challenges the power and infrastructures of big data and gives each individual the right to choose how their personal data are used [21]. Hakkila et al explored the use of wearable sensors connected to smartphones to collect personal activity and health data for wellness and suggested that these data should be stored within the MyData schema [22]. In this way, they envisioned the necessary information and awareness for individuals to pursue healthier lifestyles with personal data under their control.

However, Choe et al signaled that individuals may have difficulty in data integration and interpretation when personal data sets are collected using mobile technologies [23]. Choe et al examined the experiences of individuals wearing sensors to collect and track personal data, such as sleep patterns, heart rate and location. They report that managing, visualizing and interpreting this data is a significant barrier to its usefulness [23]. With a concern for making sense of health data, Katz et al investigated the use of apps on smartphones for diabetes self-management. They found that apps on smartphones did not support the cognitive load needed to make comparison between data and to reflect its meaning [24]. They surmised that the limited screen size of the smartphone prevented data visualization for deep engagement and sense-making [24]. Lyons et al combined the technologies of wearable monitors and mobile tablet apps in an intervention with the focused goal of increasingly physical activity in older adults [25]. They concluded that when accompanied by brief phone counselling, small increases in physical activity resulted and that there was potential for a behavior change when these technologies were merged for specific intent [25].

The literature, however, is silent on the value of small personal data sets that are manually collected, stored and manipulated in apps, and then displayed graphically on the screens of mobile tablet devices for self-management of chronic disease. Therefore, the value that this may present in the self-management of chronic disease is also unknown. The research question for this study thus asks:

Are mobile devices and associated apps useful tools for type 2 diabetes self-management?

## Methods

### Research Design

This study was conducted in a large Australian integrated primary healthcare clinic which houses an interdisciplinary team of health professionals that includes physiotherapists, radiologists, and dieticians all in one physical location. All aspects of the project have the full approval of the Human Research Ethics Committee at the University of Canberra (approval number 14-85). Digital activities for diabetes self-management were designed at a participatory design workshop attended by healthcare professionals and researchers [26]. Two of the six activities that were suggested to the research participants in the form of invitations were: (1) to record their biomeasures such as blood glucose level (BGL), weight, and blood pressure; and (2) to use diet apps to plan and record their food intake. Responses to these two invitations are discussed in this article. Apps that supported these two activities (*Glucose Wiz* for recording biomeasures, and *Food Switch*, *Easy Diet Diary* and *iCookbook Diabetic* for dietary management) were preinstalled on the iPads given to participants. However, the participants were also able to download apps of their choice from the App Store. The choice and quality of the apps was not a focus of the study. It was important to rule out lack of digital skill as a cause for nonengagement in the program. All participants attended an initial workshop where Wi-Fi and 3G enabled iPads with 16GB were distributed. The goal of this workshop was to ensure basic digital establishment and literacy. Participants were supported in setting up an email address, obtaining an Apple ID, downloading apps from the Apple store and 3G subscriber identity module (SIM) card activation. Individual consultations and online support, which included the use of the device and apps, were participant-directed. They were offered for as long as the participants perceived a need.

### Recruitment

The gifting of iPads to research participants and the intensive digital support that was made available for the duration of the study meant sample size was set at approximately 30 to conform with the project budget. Participant recruitment started on August 6, 2014 and the quota were filled by August 22, 2014. Because diabetes patients visit their General Practitioner (GP) at two-month or three-month intervals and the recruitment window would therefore not reach the full spectrum of available patients, the recruitment strategy was twofold. Firstly, there was advertising in the clinic waiting room and the ability to self-nominate. Secondly, on health professional’s recommendation, nurses could invite patients to participate by phone. Three of the initial participants, learning more about the commitment needed in the program, withdrew in the early weeks and several more patients were recruited to take their place. The final number of people with type 2 diabetes participating in the study was 28, and no specific inclusion or exclusion criteria were used in selecting participants. A summary of the demographics of participants is provided in Table 1, revealing a diverse range of socioeconomic and educational backgrounds.

**Table 1:** Demographic summary of participants (N=28).

Demographics		n (%)
**Gender**		
	Male	16 (57)
	Female	12 (43)
**Age (years)**		
	30-39	1 (4)
	40-49	4 (14)
	50-59	10 (36)
	60-69	9 (32)
	70-79	4 (14)
**Education**		
	High school	9 (32)
	Diploma or associate degree	12 (43)
	Bachelor’s degree	3 (11)
	Postgraduate degree	4 (14)
**Diagnosis**		
	< 6 months	8 (29)
	6 months to 2 years	3 (11)
	2-9 years	9 (32)
	10+ years	8 (29)

### Data Collection and Analysis

Using online questionnaires as the main method of collecting data from participants, both quantitative and qualitative data were sought. Quantitative data were collected in order to measure and establish demographics, trends and preferences, and qualitative data were collected in order to gain insight into, and understanding of, the human experience. All four online survey tools were developed iteratively and were informed by established measures of health behavior, digital literacy and digital engagement.

This article reports a secondary analysis of a subset of the data obtained in the larger study described above. It draws from the analysis of qualitative data obtained via open-ended written responses across four online surveys that were conducted over the nine-month intervention. Open-ended questions elicited information about the participants’ use and perception of the mobile device, their digital literacy, their use of apps for self-care, perceived change in their health, changes in biomeasures and the ways in which all of this was enacted.

Thematic analysis, used in this study, captures a level of patterned meaning within the data [27] and provides a theoretical freedom to approach a complex body of data and reveal themes and insights without pre-existing expectations. It is inductive in nature and “has a long tradition of use” in applied health research [28]. The qualitative data in this study, resultant of the open response survey questions, existed in structured textual form and were coded across all questions. In an iterative approach, analysis took place at the conclusion of each of the four surveys and emerging thematic concepts informed the design of the next survey. Following the coding of each survey and tentative theme construction, the four survey responses from each individual were re-examined to gain an alternate view of the data and further insight into the emerging themes. The coding was performed by a single researcher for consistency and the research team met periodically to discuss the emerging constructs.

## Results

### Overview

Results are presented as themes, which are constructs indicated by a detailed analysis of the data rather than found in the data itself [29]. The following themes emerged from the analysis of data collected in this study and reveal a logical sequence of digital engagement for self-management of diabetes. Accustomed to measuring and recording biological measures such as BGL and blood pressure, many of the research participants used the tablet device and its apps to record these measures, building a small personal data set over time. The data were then used to model and graphically display personal information that was, in turn, interpreted by participants by comparing it with other self-care data, such as dietary intake, which they had recorded using the tablet device. Throughout this article, pseudonyms are utilized when data provided by participants is used to illustrate themes and inform the reader.

### Recording Personal Data

Self-management of type 2 diabetes typically includes measuring and recording BGL, weight, and blood pressure as part of a routine of self-care [11]. The iPad proved a useful tool to support these activities:

The iPad has helped me to record blood glucose levels, diet and exercise, all of which are important for managing diabetes.

Ruth

One participant outlines the simplicity of the process:

I use this app to record my glucose readings 3 times a day. It's very simple to use - just enter the reading and it records it for you.

Lily

Leah reports that she “now takes accurate information to her doctor all the time.” She compares her recordkeeping before the iPad:

Before I had the iPad and the apps, I was trying to keep records on paper, spreadsheets on the computer, it was a mess, I would lose the paperwork, forget to record on my desktop.

Leah

At the conclusion of the program, 79% of participants were using an app to record their BGL data. It was the most frequent purpose for using apps. Table 2 shows the number of people using the pre-installed apps [30].

**Table 2:** The number of people using the pre-installed apps (N=28).

Pre-installed app name	n (%)
Glucose Wiz	12 (43)
iCookbook Diabetic	11 (39)
Food Switch	7 (25)

Medication times and dates were less commonly recorded; however, one participant, Tristan, reports that medication times and dates were his main use of the tablet in an environment where “the iPad stays on the table to remind me to take the medication.” This countered a pattern of Tristan having “missed [his] medication at least once or twice a fortnight.”

### Modelling and Visualizing the Data

With a small data set in place, the modelling capacity of the apps was discovered by participants. They were self-taught in producing varied models of their own data, which could be graphically displayed. One participant said that:

Glucose Regulator is an easy and quick app to record and graph my BGL's and makes the trends in my levels so clear.

Rachel

Participants could look at their history and patterns with ease:

There are two functions that I particularly like - once recorded you can look at your readings in a graph format over a period of days, plus it will also give you an average reading - which is great for your doctor to view too!

Lily

Claire valued the time and period of the day that was stamped with her granular BGL data:

The Glucose Wiz app ties each reading to a time and the appropriate period eg. before a meal, a random mid period reading, after a meal etc. In addition it shows the averages for before meals, after meals.

In combination with the recording and modelling capability of the apps, the visual, graphical representation of data was extremely beneficial to participants, with Ruth saying:

I like the clarity of the BGL graph.

“Visual feedback, which I have found most useful,” was important to Raymond. His results were much clearer to him in diagrammatic form. Extremely appreciative of the data visualization capability of her chosen app, this participant reports:

This app makes charts of blood pressure fluctuations, with upper and lower parameters with colour warnings. As well, this app records the time, the measurement, the arm used, body position, recent meal, weight, height, BMI, body fat, cholesterol, LDL, HDL and water consumption. It is easy to use and the charts are just like magic.

Madelyn

Charts, tables, averages and trends were shared with health professionals, the mobile tablet being acclaimed by participants for its portability and visual display of data, with Ruth saying:

It has also been beneficial for my Dr to see my levels at a glance.

### Interpreting the Data

Ryan adopted regular recording of his BGL on Glucose Wiz and “where an anomaly occurred, [he] was able to determine what [he] was eating to correct the issue.” With a baseline for BGL presented as graphical information, he could watch for spikes and consider what he had eaten at that time. In this way, Ryan reported a reduction in BGL, weight loss and increased fitness at his next consultation with his healthcare professional.

Participants reported that monitoring the data was an important activity in self-management and when irregularities were noted, explanations were sought. Raymond monitors his circumstances:

The visual feedback I get from Glucose Wiz enables me to keep track of my progress.

Keith looks for changes in his BGL data and maps them to his diet for the day

I am able to record by BGL test on the iPad, track the test over a week to a month, see what I have been eating that day and what I need to move on.

This monitoring was made easier by the visualization of the data. Another participant reported being able to customize her own meals to avoid insulin spikes by mapping her BGLs to a record of food eaten.

The mobile tablet device presents self-management data with graphical clarity. It provides chronological and accurate records for diabetes self-management for this participant:

While you may have an idea of how you are managing your diabetes, the graphical information presented in the app clearly shows how well you are in fact managing.

Anna

The personal data sets and visualizations make for realistic assessment and decisions by an individual. For Raymond, his own data collected via apps “helps me to keep on track, and to get back on track when I need to.” Keith notes improved self-management and its outcomes:

I check my BGL more, I check my weight more, monitor my diet better. My blood levels are staying more normal than over a year ago.

The following participant describes an improvement in self-management that has developed over time and increased his knowledge and routine:

When I first used the device to record sugar level, I kept monitoring my levels frequently to get my level down. I feel over time, I have developed a routine of diet and exercise.

Jackson

Jackson says that he has learned a lot in this way, and that “it has changed the way that I live.” Emily has been assisted with “more tools at my disposal and a greater ability to track various things.” Because participants were invited to engage digitally rather than required to participate in any specific way, the uptake of the iPad as a management tool varied from minimal to a comprehensive engagement such as Madelyn’s:

I track as many of my metabolic markers as I can, using various apps. I can see in my charts how my BGL fluctuates throughout the day or the month and also heed warnings about hypertension.

### Empowering and Improving Health

The iPad and apps were regarded as empowering tools that enabled a sense of control over the participants’ health. Kaylee said that:

I feel more in control with the help of the apps in the iPad in managing my diabetes.

Menon claimed that a sense of control is an essential component of health empowerment [15]. According to one participant, Claire:

The greatest feeling of good management is the feeling of well-being and energy you get when blood sugars are at acceptable levels and weight is under control.

Recognizing from the depiction of his BGLs that his diabetic management was “not up to scratch” on a recent cruise, Ryan discussed managing diet when travelling and the importance of portion size with his doctor and was able take control once more.

Cause and effect are demonstrated to Raymond and he becomes more aware of the consequences of his choices:

The graphical picture on charts from Glucose Wiz enabled me to see that good results do happen, when you are doing the right thing.

Rachel makes notes on her app “if I do have any unusual results.” With an existing diagnosis of type 2 diabetes, she noted that her BGLs were increasing in spite of her vigilant lifestyle and monitoring. Using the iPad to research her symptoms, she suggested to her doctor that she should be tested for a rare form of diabetes. She was tested and found to have latent autoimmune diabetes in adults and was treated accordingly.

Madelyn reported that her visits to the doctor were more interesting with the records that she had made. She displayed BGLs, blood pressure, weight, BMI (body mass index), body fat percentage, hemoglobin A_1c_ (HbA_1c_) and hydration “in impressive chart form of chronological order.” Madelyn’s self-management using her personal small data brought about change in her health:

My weight is now around 69kg [from 125kg], my BGL are in the normal range and I have an iPad which has a complete record of the transformation.

Using the Glucose Wiz app to record her weight, medication and BGLs, Leah was feeling very unwell and dramatically losing weight, and she said that “my BGLs were unpredictable and rising from week to week.” Leah could see a contradiction between her strict diet and her BGLs. She handed her data set to her GP:

I gave my iPad to the doctor, it was due to my iPad that I was diagnosed with type 1 diabetes and sent to a specialist.

Leah continues to monitor her biomeasures on the iPad in the management of her newly diagnosed condition. For most participants who adopted mobile management, however, the shift in their health was less dramatic. Jackson describes his health situation having adopted the iPad as a self-management tool as “close to normal BGL and normal blood pressure – Doctor was happy.”

## Discussion

### Principal Findings

This article reports successful personal health data collection, curation, visualization and use by research participants, which is counter to the studies of personal data collection by Choe et al who reported small individual data sets captured by wearable monitors as difficult to manage and to understand [23]. We suggest that the point of difference of our study to that of Choe et al is the individual’s purposeful measurement and recording of each item of data. Measures were taken and entered into apps that support data storage and display. This differs from tracking devices that automatically read and store a large amount of data that proves more difficult to interpret and convert to information that is useful to its owner.

This study also sits in contrast to the big data phenomenon that is prevalent in many societal contexts including health. Big data can be unstructured data in a variety of formats such as video and audio and is produced in vast and rapid streams. It is complex data that is used to establish trends and solve complex, population-wide problems [19]. As society grapples with the extent of a digital revolution that is both ill-defined and full of potential, it is important to not ignore the small, personal, isolated, yet beneficial collections of data. This study is novel in its attention to small independent digital data sets, collected by individuals for self-care of chronic illness and adds nuanced and humanistic knowledge to digital health.

This intervention places data-driven modelling and resultant visual output in the hands of an individual, empowering their management of their own disease. With a small, personal data set in place, participants in this study engaged in the modelling capability of their apps, producing averages over varying time periods, trends and patterns.

Participants embraced the app functionality that allowed them to visualize their data in graphs and charts with embedded alerts for any extremes in the data. Data visualization is a form of visual communication that seeks to improve human comprehension of granular data [31]. Uyan Dur added that the value of data visualization is that it is based on measurable statistical data, that it provides a picture of numeric data that could otherwise be difficult to comprehend [31].

Data visualization allows its viewer to more readily interpret the data and to integrate it into problem solving activities. “Visualization of information ensures the ability to see events and connections between them in new and different ways and to reveal other invisible patterns” [31]. This was evident with the participants in this study as their BGL graphs revealed the effect of inadvisable dietary intake. Importantly for several participants, the visualization of their data led to them questioning all of the available information about their condition. Anomalies were noticed to the extent that individuals contacted their doctor and new diagnoses and treatment plans were made. The benefit of data visualization was improved cognition, and at times a corresponding shift in behavior.

The iPad tablet screen size is considered ideal for manipulating data and is of a quality that is easily viewed and comprehended. It was preferred by the participants to an iPhone with identical apps because its size supported the dexterous and visual capability of its user. This is in keeping with the study of Katz et al who found a smartphone too small for the sensemaking of health data in diabetes self-management [24]. The apps, both pre-populated and chosen by participants, provided intuitive use and participants moved through recording, modelling, visualizing and interpreting their data without barriers. Individuals were empowered to produce varied models of their behaviors and biomeasures and immediate feedback was available via visual display of patterns, trends and scenarios. The result of this was improved participant cognition and discernment, with Ruth saying:

Being able to record my blood glucose levels and see them on a graph, or in red if they are high, has made me want to keep them within normal limits.

Choe et al [23] described the ideals of personal health informatics thus:

That through knowledge of one’s data, it becomes possible to reflect on one’s activities, make self-discoveries, and use that knowledge to make changes.

This study exposes the potential of mobile tablets and apps as tools for the establishment, visualization and interpretation of small personal data sets for diabetes self-management.

Clinicians are increasingly looking to enhance consumer enablement in chronic conditions such as diabetes mellitus [32]. The use of mobile tablet devices as in this study is likely to facilitate an increased enablement of individual consumers, which means an improvement to the extent to which they understand their health conditions and have the confidence, skills, knowledge and ability to manage their health and wellbeing. This in turn may translate into improved clinical outcomes and experience of care.

This article reports a qualitative research approach that examines human behavior in a complex intervention of technology, digital engagement, primary health care and self-management of chronic disease. It contributes to the health of society by delivering a rich, theoretical description of successful use of mobile devices in diabetes self-management from a humanistic perspective [33]. The study could be recontextualized and applied in similar settings and with other chronic illnesses.

The limitations of this study include the collection of qualitative data via an online survey tool. This approach was taken because of the longitudinal nature of the study that collected data on four occasions. Yet, it prevented an interaction between researcher and participant that could have probed deeper into some of the questions that were asked with follow-up queries.

### Conclusion

This article draws attention to the potential of mobile tablet devices for the self-management of type 2 diabetes. It illuminates the concept of small, personal health data and its collection and management by an individual to improve their health. Once proficient with measuring, entering, and storing biomeasures that support self-management, individuals turn their attention to the modelling, visual display and interpretation of their own health data. In this, an accessible tablet device provides a superior output screen for diagrammatic health information. Personal digital health activities shift from the consumption of text and static diagrams to dynamic data visualization of personal measures, from which insight results and behavior can change.

## Abbreviations

BGL: blood glucose level
BMI: body mass index
GP: general practitioner
HbA_1c_: hemoglobin A_1c_
PDA: personal digital assistant
SIM: subscriber identity modules

## References

[1] Weymann N, Härter M, Dirmaier J (2016). Information and decision support needs in patients with type 2 diabetes. Health Informatics J.

[2] Burford S, Park S (2014). The impact of mobile tablet devices on human information behaviour. Journal of Documentation.

[3] World Health Organization (2011). Global Observatory for eHealth Series (Vol 3).

[4] Shaw RJ, Steinberg DM, Zullig LL, Bosworth HB, Johnson CM, Davis LL (2014). mHealth interventions for weight loss: a guide for achieving treatment fidelity. J Am Med Inform Assoc.

[5] Whittaker R, Merry S, Dorey E, Maddison R (2012). A development and evaluation process for mHealth interventions: examples from New Zealand. J Health Commun.

[6] Burns M, Begale M, Duffecy J, Gergle D, Karr C, Giangrande E, Mohr D (2011). Harnessing context sensing to develop a mobile intervention for depression. J Med Internet Res.

[7] Zarghom S, Di Fonzo D, Leung F (2013). Does Socioeconomic Status Affect Patients' Ease of Use of a Touch-Screen (iPad) Patient Survey?. Interact J Med Res.

[8] Hunt CW, Sanderson BK, Ellison KJ (2014). Support for diabetes using technology: a pilot study to improve self-management. Medsurg Nurs.

[9] Drayton K (2013). How mobile technology can improve healthcare. Nursing Times; 109(11).

[10] National Diabetes Strategy Advisory Group (NDSAG).

[11] Coyle ME, Francis K, Chapman Y (2013). Self-management activities in diabetes care: a systematic review. Aust Health Rev.

[12] Funnell M, Anderson R (2004). Empowerment and Self-Management of Diabetes. Clinical Diabetes.

[13] Meetoo D, Gopaul H (2005). Empowerment: giving power to people with diabetes. J Diabetes Nurs.

[14] Rafael AR (1995). Advocacy and empowerment: dichotomous or synchronous concepts?. ANS Adv Nurs Sci.

[15] Menon ST (2002). Toward a model of psychological health empowerment: implications for health care in multicultural communities. Nurse Educ Today.

[16] Samoocha D, Bruinvels DJ, Elbers NA, Anema JR, van der Beek AJ (2010). Effectiveness of web-based interventions on patient empowerment: a systematic review and meta-analysis. J Med Internet Res.

[17] Burford S, Park S, Carpenter M, Dawda P, Burns J (2016). Digital engagement, self-management, and shifting the locus of control: a mHealth program for people with type 2 diabetes. https://ieeexplore.ieee.org/document/7427604.

[18] Gimbel R, Shi L, Williams JE, Dye CJ, Chen L, Crawford P, Shry EA, Griffin SF, Jones KO, Sherrill WW, Truong K, Little JR, Edwards KW, Hing M, Moss JB (2017). Enhancing mHealth Technology in the Patient-Centered Medical Home Environment to Activate Patients With Type 2 Diabetes: A Multisite Feasibility Study Protocol. JMIR Res Protoc.

[19] Mayer-Schonberger V, Cukier K (2013). Big Data.

[20] Keen J (2014). Digital health care: cementing centralisation?. Health Informatics J.

[21] Poikola A, Kuikkaniemi K, Honko H (2015). Finnish Ministry of Transport and Communications.

[22] Hakkila J, Alhonsuo M, Virtanen L, Rantakari J, Colley A, Koivumaki T (2016). MyData approach for persona health: a service design case for young athletes.

[23] Choe E, Lee N, Lee B, Pratt W, Kientz J (2016). Understanding quantified selfers? practices in collecting and exploring personal data.

[24] Katz D, Dalton N, Holland S, O'Kane A, Price B (2016). Questioning the reflection paradigm for diabetes mobile apps.

[25] Lyons EJ, Swartz MC, Lewis ZH, Martinez E, Jennings K (2017). Feasibility and Acceptability of a Wearable Technology Physical Activity Intervention With Telephone Counseling for Mid-Aged and Older Adults: A Randomized Controlled Pilot Trial. JMIR Mhealth Uhealth.

[26] Burford SJ, Park S, Dawda P, Burns J (2016). Participatory research design in mobile health: Tablet devices for diabetes self-management. CAM.

[27] Braun V, Clarke V (2006). Using thematic analysis in psychology. Qualitative Research in Psychology.

[28] Hansen E (2006). Successful Qualitative Health Research: A practical introduction.

[29] Morse J, Field P (1995). Qualitative Research Methods for Health Professionals(2nd ed.). Sage: Thousand Oaks, CA.

[30] Park S, Burford S, Hanlen L, Dawda P, Dugdale P, Nolan C, Burns J (2016). An integrated mHealth model for type 2 diabetes patients using mobile tablet devices. JournalMTM.

[31] Uyan DI (2014). Data visualization and infographics in visual communication design education at the age of information. Journal of Arts and Humanities.

[32] Agency for Clinical Innovation (2018). Consumer Enablement Guide 2018.

[33] Morse JM (2006). Reconceptualizing qualitative evidence. Qual Health Res.

